# Increased risk of hepatocellular carcinoma after cholecystectomy

**DOI:** 10.1038/bjc.2011.181

**Published:** 2011-05-24

**Authors:** J Lagergren, F Mattsson, H El-Serag, H Nordenstedt

**Affiliations:** 1 Upper Gastrointestinal Research, Department of Molecular Medicine and Surgery, Karolinska Institutet, Norra Stationsgatan 67, Level 2, 171 76 Stockholm, Sweden; 2 King's College London, London, UK; 3 Michael E DeBakey Veterans Administration Medical Center and Baylor College of Medicine, Houston Center for Quality of Care and Utilization Studies, Houston, TX, USA

**Keywords:** liver cancer, gall bladder, epidemiology, risk

## Abstract

**Background:**

The association between gall bladder removal (cholecystectomy) and hepatocellular carcinoma warrants investigation. An increased intrahepatic bile duct pressure following cholecystectomy might cause chronic inflammation in the surrounding liver tissue, which might induce cancer development.

**Methods:**

A nationwide Swedish population-based cohort study in 1965–2008 included 345 251 patients undergoing cholecystectomy because of gallstone. The number of observed hepatocellular carcinoma cases was divided by the expected number, calculated from the corresponding background Swedish population, thus providing standardised incidence ratios (SIRs) with 95% confidence intervals (CIs).

**Results:**

During follow-up of 4 854 969 person-years, 333 new cases of hepatocellular carcinoma were identified, rendering an overall increased risk (SIR 1.24, 95% CI: 1.11–1.38). The risk increased with longer follow-up (*P* for trend=0.003). Among patients who underwent cholecystectomy 30–43 years earlier, SIR was 2.00 (95% CI: 1.32–2.87). The results were similar after exclusion of 15 634 patients with any recorded risk factor, that is, diabetes, obesity, hepatitis, liver cirrhosis, alcoholism, or blood transfusion.

**Conclusion:**

Cholecystectomy might be associated with a long-term increased risk of hepatocellular carcinoma.

This study addresses the hypothesis that surgical removal of the gall bladder (cholecystectomy) for gallstone disease is associated with an increased risk of developing hepatocellular carcinoma. Cholecystectomy is one of the most common surgical procedures performed in the West. Hepatocellular carcinoma has the fifth highest incidence of any cancer globally and is the third most common reason for cancer death globally (El-Serag and Rudolph, 2007). Its incidence is increasing in the United States (McGlynn *et al*, 2001). However, in Sweden, the incidence is lower (about 3 per 100 000 persons and year). The only established risk factors for hepatocellular carcinoma are diabetes, obesity, hepatitis, liver cirrhosis, aflatoxin, and alcoholism (El-Serag and Rudolph, 2007). The relation between cholecystectomy and hepatocellular carcinoma merits investigation, since an association is biologically plausible. Cholecystectomy is typically followed by increased pressure in and dilation of the intrahepatic bile ducts (Tanaka *et al*, 1984), which in turn might cause chronic inflammation in the surrounding liver tissue. Such inflammation could induce proliferation of the liver cells (hepatocytes) and cause hepatocellular carcinoma (El-Serag and Rudolph, 2007). A cohort study from the Danish National Registry found a possible increase in risk of unspecified primary liver cancer in patients who had undergone cholecystectomy, while no increase was found in a cohort study from the United Kingdom (Chow *et al*, 1999; Goldacre *et al*, 2005). However, these studies were unable to evaluate long-term effects and made no distinction between the histological types hepatocellular carcinoma and intrahepatic cholangiocarcinoma. Thus, no previous study has addressed the specific relation between cholecystectomy and hepatocellular carcinoma. We conducted a large, population-based cohort study with long-term follow-up to clarify this potential association.

## Patients and methods

As described in detail elsewhere (Freedman *et al*, 2001), patients undergoing cholecystectomy because of gallstone disease were identified through the Swedish Patient Registry, which contains data on 99% of all hospitalisations and surgical procedures performed in Sweden since 1965 (Swedish Board of Health and Welfare, 2009). Each cohort member was followed up until the first occurrence of any cancer diagnosis, death, emigration, or until the end of the study period (31 December 2008). Information on cancer diagnosis was obtained from the Swedish Cancer Registry, which was initiated in 1958 and has an at least 98% completeness rate (Barlow *et al*, 2009). The tumour evaluated was primary liver cancer of the hepatocellular type (code 155.0 and histological code 066 according to the International Classification of Diseases, version 7). Information on dates of death and emigration was obtained by referral to the Swedish Total Population Registry, which is 100% complete and is constantly updated. All person-time and cancers identified during the first year of follow-up were excluded to allow a minimum latency interval between exposure and outcome and to avoid bias due to earlier detection of non-symptomatic tumours. The personal identity number, a unique identifier for each resident in Sweden, allowed each cohort member to be tracked between the registries (Ludvigsson *et al*, 2009). Standardised incidence ratio (SIR), that is, the ratio of the observed to the expected number of cases, was used to estimate relative risk. The expected number of cases was calculated by multiplying the observed person-years by age in 5 year groups, gender, and calendar years-specific cancer incidence rates. These rates were derived from the entire Swedish population through the Swedish Cancer Registry. Confidence intervals (CIs) of the SIRs were calculated on the assumption that the observed number of cancer cases followed a Poisson distribution (Breslow and Day, 1987). A test for trend with increased calendar time was performed (Breslow and Day, 1987). The regional ethics committee in Stockholm approved the study.

## Results

The cholecystectomy cohort included 345 251 people who were followed up for an average of 15.1 years (range 1–43 years). Together, they contributed 4 854 969 person-years at risk. The mean age at cholecystectomy was 52 years (50 in women and 57 in men), and 67% of patients were women. During the entire follow-up period, 333 new cases of hepatocellular carcinoma were identified after the first year. There was a statistically significant increased overall risk of hepatocellular carcinoma compared with the corresponding background population (SIR 1.24, 95% CI: 1.11–1.38; Table 1). During the first decade after cholecystectomy, no significantly increased risk was found, but thereafter the risk gradually increased with longer follow-up time (*P* for trend=0.003). Among patients who had undergone cholecystectomy at least 30 years earlier, the SIR of hepatocellular carcinoma was increased two-fold compared with the corresponding background population (SIR 2.00, 95% CI: 1.32–2.87). The association was found only in younger age groups, and was stronger in females (Table 1).

Analyses were repeated after excluding 15 634 patients from the cohort who had any recorded diagnosis of diabetes, obesity, hepatitis, liver cirrhosis, or alcoholism before the cholecystectomy, or blood transfusion, as these conditions could confound the association between cholecystectomy and hepatocellular carcinoma. Similar results were observed; the increase in SIRs with increasing exposure time after cholecystectomy was as evident as in the total cohort (Table 2). Among patients with the longest exposure time (i.e., those who had a cholecystectomy at least 30 years earlier) the SIR was 1.93 (95% CI: 1.27–2.81; Table 2).

## Discussion

This study provides evidence suggesting there is an increased long-term risk of developing hepatocellular carcinoma among patients who have undergone cholecystectomy.

The population-based cohort design, the high validity and completeness of the databases used, the large size of the cohort, and the long and complete follow-up of all participants are among the methodological advantages of the study. Confounding is a particular threat to observational research. Potential confounding by age, sex, and calendar year was adjusted for in the study design. Conditions related with hepatocellular carcinoma, that is, hepatitis B and C infection, alcohol consumption, liver cirrhosis, obesity, diabetes mellitus, and blood transfusion (El-Serag and Rudolph, 2007), should not act as confounders in the study since we evaluated confounding by these risk factors by excluding patients with a recorded diagnosis of at least one of these conditions, and found no support for confounding being responsible for the association. However, this exclusion may not be complete, since all patients with these conditions might not be hospitalised and hospitalisation might change over time. Thus, some level of residual confounding from these factors cannot be excluded. Bias due to earlier detection of hepatocellular carcinoma as part of the cholecystectomy was unlikely as there was a lack of association initially after surgery. Moreover, we excluded the first year after cholecystectomy to prevent detection bias. The risk of tumour misclassification was negligible, since only primary liver cancers with a histologically verified hepatocellular carcinoma were considered in the study. Finally, chance is an unlikely explanation for the association, because of the large sample size and the dose–response effect seen with increased latency time. Thus, the finding should not be dismissed for methodological reasons.

The lack of association seen during the first 10 years following exposure (cholecystectomy) and the gradually increasing incidence of invasive hepatocellular carcinomas after 10 years argue in favour of a true association. To the best of our knowledge, no previous study has addressed a possible relation between cholecystectomy and hepatocellular carcinoma. Therefore, the finding needs to be confirmed by future research before conclusions can be drawn and it is too early to consider any potential clinical recommendations. Two previous cohort studies investigated cholecystectomy in relation to primary liver cancer without specifying the histological type (Chow *et al*, 1999; Goldacre *et al*, 2005). A Danish study found a statistically non-significant increased risk, while a UK study found no increased risk after excluding the first 2 years of follow-up. However, since the number of hepatocellular carcinoma cases was limited in these two studies (48 and 38, respectively) and the follow-up time was short, it was not possible to evaluate any long-term effects.

If the association is true, a potential pathway might involve chronic inflammation. Chronic inflammation is an accepted carcinogenic mechanism for several types of cancer, including hepatocellular carcinoma, and cell proliferation is present in most liver disease as a consequence of chronic inflammation. Cholecystectomy is typically followed by dilation of the common biliary duct and a rise in bile duct pressure, both of which might increase the risk of chronic inflammation and cell proliferation of the hepatocytes.

More research is required to establish any true association between cholecystectomy and hepatocellular carcinoma, but assuming that a two-fold increased risk is true, the absolute risk would increase from about 3 per 100 000 persons and year to 6 per 100 000 persons and year in Sweden. The individuals’ risk is, therefore, still limited and the results should be used to influence clinical decision-making in patients in need for cholecystectomy.

In conclusion, this population-based cohort study of 345 251 people with nearly 5 million person-years at risk, with follow-up of up to 43 years, indicates that cholecystectomy is associated with an increased risk of hepatocellular carcinoma.

## Figures and Tables

**Table 1:** Risk of hepatocellular carcinoma among 345 251 people patients who had a gall bladder removal (cholecystectomy) during the period 1965–2008 in Sweden, expressed as standardised incidence ratio (SIR) with 95% confidence interval (CI)

		**Hepatocellular carcinoma**	
**Characteristic**	**Person-years**	**Number**	**SIR (95% CI)**
All	4 854 969	333	1.24 (1.11–1.38)
			
*Gender*			
Male	1 418 031	186	1.18 (1.01–1.36)
Female	3 436 938	147	1.34 (1.13–1.57)
			
*Age at entry (years)*			
<60	3 654 343	176	1.46 (1.25–1.69)
60–69	785 219	100	1.20 (0.98–1.46)
⩾70	415 407	57	0.89 (0.68–1.16)
			
*Years after cholecystectomy*			
1–4	1 194 909	60	1.10 (0.84–1.41)
5–9	1 145 868	63	1.02 (0.78–1.30)
10–19	1 498 022	113	1.24 (1.02–1.48)
20–29	762 266	69	1.49 (1.16–1.89)
⩾30	253 904	28	2.00 (1.32–2.87)

All person-time and cancers accrued during the first year of follow-up were excluded.

**Table 2:** Risk of hepatocellular carcinoma among 329 617 people patients who had a gall bladder removal (cholecystectomy) during the period 1965–2008 in Sweden after exclusion of 15 634 persons with any recorded diagnosis of diabetes, obesity, hepatitis, liver cirrhosis, or alcoholism before the cholecystectomy, or possible blood transfusion during or after the cholecystectomy

		**Hepatocellular carcinoma**	
**Characteristic**	**Person-years**	**Number**	**SIR (95% CI)**
All	4 732 507	298	1.14 (1.02–1.28)
			
*Gender*			
Male	1 368 768	165	1.08 (0.92–1.26)
Female	3 363 739	133	1.23 (1.03–1.46)
			
*Age at entry (years)*			
<60	3 570 621	163	1.38 (1.17–1.60)
60–69	763 071	89	1.10 (0.88–1.35)
⩾70	398 814	46	0.75 (0.55–1.00)
			
*Years after cholecystectomy*			
1–4	1 149 673	46	0.89 (0.65–1.18)
5–9	1 111 908	50	0.84 (0.62–1.10)
10–19	1 466 894	108	1.21 (0.99–1.45)
20–29	752 036	67	1.47 (1.14–1.87)
⩾30	251 996	27	1.93 (1.27–2.81)

All person-time and cancers accrued during the first year of follow-up were excluded.
